# Bibliographical Notices

**Published:** 1844-06-15

**Authors:** 


### BIBLIOGRAPHICAL NOTICES.

*Traité de Matière Médicale et de Thérapeutique appliquée a chaque Maladie en particulier:* par Le Docteur F. FOY, Pharmacien in chef de l'hôpital St. Louis. Tome 1re. Matière Médicale. Tome 2de. Thérapeutique appliquée à chaque Maladie en particulier. 8vo. pp. 628 & 713. Paris, 1843.

*Formulaire des Médecins Praticiens,* contenant. 1. les Formules des Hôpitaux civils et militaires Français et étrangers; 2e. l'examen et l'interrogation des Malades; 3e. un Mémorial raisonné et thérapeutique; 4e. les Secours à donner aux empoisonnés et aux asphyxiés; 5e. la Classification des Médicaments d'apres leurs effets thérapeutiques; 6e. un Tableau des substances incompatibles; 7e. l'Art de formuler; par M. le Docteur Foy, Pharmacien en chef de l'Hôpital Saint Louis. 4ème. édit. augmentée d'un supplement pour les médicaments nouveaux et les Formules nouvelles et d'une Table alphabétique des Auteurs et des Matières. 18mo. pp. 396. 1844.

*Annuaire de Thérapeutique de Matière Médicale de Pharmacie et de Toxicologie pour* 1843, contenant le Resumé des Travaux Thérapeutiques et Toxicologiques publiés en 1842, et les Formules des Médicaments nouveaux, suivi d'un Mémoire sur la Digestion lu à l'Académie des Sciences, par MM. Bouchardat et Sandras: par le Dr. A. Bouchardat, Professeur agrégé de la Faculté de Médecine de Paris, &c. &c. 24mo. pp. 302. Paris, 1843.

Were we to judge of the amount of matter contained in these volumes by their respective titles, we should judge most erroneously, as we often do in such cases, and be seduced by the physiognomy, or the appearance of that which is without, to form a most erroneous estimate of the amount of that which is within. Yet are none of them deficient in extent of information. Each is sufficiently well adapted for its object, and each forms a useful addition to the therapeutical portion of any practitioner's library.

We have had some rich accessions on the subject of Materia Medica of late years. Pereira's work may be placed at the head, for extent and variety of information on the natural and chemical history of the substances used in medicine. Christison's Dispensatory, and our own excellent Dispensatory of the United States by Professors Wood and Bache, are admirable of their kind, but necessarily restricted by the very object which their authors had in view. Another edition of Thomson's Materia Medica and Therapeutics has added to the stock of valuable English books. It has not been reprinted in this country. Trousseau and Pidoux have given us one of the best works on the subject that have emanated from the Parisian press. The general Therapeutics and Materia

Medica of Dr. Dunglison has been designed for the use of the medical schools more especially, and has the important feature—not peculiar to it, however—that the principal attention has been directed to the articles of the Materia Medica as *medicines;* for a minute knowledge of the commercial and natural history of drugs, and in many cases of their mode of preparation on the large scale, although important to the apothecary and dealer in drugs, is less so to the practitioner of medicine than a thorough acquaintance with their uses, modes of administration and therapeutical properties ; and *last,* and in our judgment,—and, we believe, in the impartial judgment of all, not merely of "rival authors of works of a similar character"—the *least* of the productions which we shall specify, is the Dictionary of Materia Medica and Practical Pharmacy, of Mr. Brande—a mere *rifacimento* of the Manual of Pharmacy of the same author, which, notwithstanding all the patching and furbishing—or, to use the language of a special calling—in spite of the casing and jewelling which it has experienced at the hands of Dr. Bell, does not seem to have *gone* better on this side of the Atlantic than on the other, and has, we fear, irretrievably *stopped,* in spite of the *spring* which it was expected to receive from its new *dial-plate*—"*A Practical Dictionary of Materia Medica,* &c., on the basis of Brande, by John Bell, M. D., &c. &c."

But these are not all the works in the department of Materia Medica and Therapeutics that have appeared of late years. They are, however, sufficient to show, that the stock is rising, and that there are more speculators than formerly. The first and larger work of M. Foy sufficiently exhibits that—as might, by the by, be expected from his position of *pharmacien* at the Hôpital St. Louis—he knows more of pharmacy than he does of therapeutics. This is significantly demonstrated by the classification of therapeutical agents which he adopts. We do not place much stress upon such classifications, yet we think we may infer from them, whether an author has sound or sufficient views of therapeutics. What shall we say of M. Foy, who distributes all medicines after their therapeutical effects, into *Tonics, Depletives, Sedatives,* (*Calmants,*) *Evacuants,* and *Specifics,*—ranking in the last class,—*Febrifuges, Antisyphilitics, Antiherpetics, Antipsorics, Antiscrophulous* agents, *Antidotes* and *Vaccine!*

Under the separate articles we have, as in the case of phosphorus,—which we take at random,—its definition and description, sophistications, mode of preparation, uses, pharmaceutical preparations, doses, antidotes and history—given with such brevity as to be often somewhat obscure. Repeatedly, indeed, might M. Foy justly exclaim, "*brevis esse laborans obscurus fio.*" The first volume, as the title imports, investigates the nature and properties of the articles of the Materia Medica ; the second is a work "on Therapeutics applied to each disease in particular." It comprises, first, a definition of Therapeutics; 2dly, an historical, and, of course, very succinct history of the systems, doctrines and methods, which have prevailed in the science ; of the progress of therapeutics, with observations on the certainty of medicine, homœopathy, hydropathy, &c. ; 3dly, an abridged formula of the mode of examining patients; and 4thly, an inquiry into the modifications borrowed by medicine from physics, pharmacy, hygiène and dietetics, (quære, is not dietetics a branch of hygiène?) "and which could not figure amongst medicines properly so called."

The lengthened title of the "*Formulary for Practical Physicians*" of M. Foy amply explains its object. The fact of its being in its 4th edition shows that it has found favor with his countrymen. It is, doubtless, a useful volume, but we confess we are not partial to formularies. They are too apt to lead to undue confidence in drugs and in special prescriptions, to impel the practitioner to *prescribe* rather than to *think,* and hence we have always deplored to hear of such preparations as *Scudamore's Mixture, Hope's Mixture,* and the different pills and lozenges that bear the names of distinguished physicians. Their whole tendency is to empiricism.

M. Bouchardat's little *Annual* differs from the last, and resembles more in object, but not in scope, *Braithwaite's Retrospect.* This is the third volume that has appeared, and the materials for it have been obtained from the medical and pharmaceutical publications of the year 1842. The list of journals and proceedings of societies which the author has consulted and cited in his preface, is considerable, and indicates great industry on his part.

The only thing which may be regarded as out of place is the "*Essay on Digestion,*" by MM. Bouchardat and Sandras. This, interesting as it is, cannot, by any torsion, be forced into a "contribution to therapeutics." The excuse of M. Bouchardat will be, that in a work of his own he was justifiable in citing his own productions. We are willing to cede the general principle, notwithstanding the objections that have been urged by our friend, the editor of the *Bulletin of Medical Science,* (May, 1844, and June, 1844, p. 216.) Yet we think we could readily shew more than one example, in which *his* precept and *his* practice were not in accordance. We happen to have before us at this very time, for a different purpose, Combe on Infancy, with notes by the Editor of the "Bulletin," in which the references to *his* "Health and Beauty," *his* "Journal of Health," and *his* "Baths and Mineral Waters," are certainly "by no means scant;" nor do we find fault with him for this, even although the readers of his notes might "at first suppose, that the other works by the editor are the chief or only available authorities on the several branches of which they treat—an inference which we can hardly imagine was meant to be made."

It would have been wise, however, we think, in the case of the work under notice, if M. Bouchardat had resisted the parental impulse which led him to *cradle* his offspring where he has done; an opportunity would doubtless ere long have presented itself for permitting it to *sleep,* if it have not already slept, elsewhere.

